# Gut Microbiota-Dependent Metabolite Trimethylamine N-Oxide Contributes to Cardiac Dysfunction in Western Diet-Induced Obese Mice

**DOI:** 10.3389/fphys.2017.00139

**Published:** 2017-03-21

**Authors:** Kui Chen, Xiaoqian Zheng, Mingchen Feng, Dongliang Li, Hongqi Zhang

**Affiliations:** ^1^Department of Anesthesiology, Jining NO.1 People's HospitalJining, China; ^2^Outpatient Department, Jining NO.1 People's HospitalJining, China; ^3^Department of Critical Care Medicine, Jining NO.1 People's HospitalJining, China; ^4^Department of Anesthesiology, Qilu Hospital, Shandong UniversityJinan, China

**Keywords:** western diet, trimethylamine N-oxide, cardiac function, inflammation, fibrosis

## Abstract

Excessive consumption of diets high in sugars and saturated fat, frequently known as western diet (WD), may lead to obesity and metabolic syndrome. Recent evidence shows that WD-induced obesity impairs cardiac function, but the underlying mechanisms are not fully understood. Trimethylamine N-oxide (TMAO), a gut microbiota-dependent metabolite of specific dietary nutrients, has emerged as a key contributor to cardiovascular disease pathogenesis. We tested the hypothesis that elevated circulating TMAO levels contribute to cardiac dysfunction in WD-induced obesity. CD1 mice were fed a normal diet (ND) or a WD, without or with 1.0% 3,3-Dimethyl-1-butanol (DMB, an inhibitor of trimethylamine formation) in drinking water for 8 weeks. Compared with mice fed a ND, mice fed a WD showed a significant increase in body weight and dyslipidemia, and had markedly higher plasma TMAO levels at the end of the feeding protocol. Echocardiography revealed that cardiac systolic and diastolic function was impaired in mice fed a WD. DMB treatment had no effects on body weight and dyslipidemia, but significantly reduced plasma TMAO levels and prevented cardiac dysfunction in mice fed a WD. In addition, mice fed a WD had elevated expression of pro-inflammatory cytokines tumor necrosis factor-α and interleukin IL-1β, decreased expression of anti-inflammatory cytokine IL-10, and increased interstitial fibrosis in the hearts, all of which were prevented by DMB treatment. Notably, DMB treatment also reduced plasma TMAO levels in mice fed a ND but did not alter other parameters. These results suggest that consumption of a WD increases circulating TMAO levels, which lead to cardiac inflammation and fibrosis, contributing to cardiac dysfunction. Interventions that reduce circulating TMAO levels may be a novel therapeutic strategy for prevention and treatment of WD-induced cardiac dysfunction.

## Introduction

The prevalence of obesity is now recognized worldwide as a major health problem and has reached epidemic proportions, affecting both genders and all ages (Flegal et al., 2012; Head, 2015). Large epidemiological studies have conclusively showed that obesity is associated with increased mortality mostly caused by augmented risk of cardiovascular disease (Prospective Studies et al., 2009). Obesity is a major risk factor for heart failure (Kenchaiah et al., 2002; Baena-Díez et al., 2010). Several cohort studies of heart failure patients have revealed that nearly 35% of those patients are obese and that 60% are overweight (Gustafsson et al., 2005). Although it is clear that genetic factors and sedentary lifestyles play pivotal roles in the rising prevalence of obesity, dietary factors, particularly the consumption of foods rich in sugars and saturated fat, frequently known as western diet (WD), is thought to be a major risk factor for development of obesity (Sahoo et al., 2015). Experimental studies have recently shown that WD-induced obesity in mice impairs cardiac function (Carbone et al., 2015; Kesherwani et al., 2015), but the underlying mechanisms are not fully understood.

Emerging evidence reveals that the gut microbiota is implicated in the pathogenesis of cardiovascular disease (Tang and Hazen, 2014). Animal and human studies have demonstrated a meta-organismal pathway in which the gut microbiota plays an obligatory role in the metabolism of trimethylamine (TMA) containing nutrients, such as L-carnitine, choline, and betaine (Wang et al., 2011; Wang Z. et al., 2014; Boutagy et al., 2015a). The metabolism of these constituents produces TMA, which is readily absorbed and travels via the portal circulation to the liver, where it is oxidized by hepatic flavin monooxygenase 3 (FMO3) to trimethylamine-N-oxide (TMAO; Wang et al., 2011; Wang Z. et al., 2014; Boutagy et al., 2015a). Elevated TMAO levels has recently been shown to be independently associated with increased risk of adverse cardiovascular outcomes, including heart attack, stroke, and death risk (Tang and Hazen, 2014, 2017; Tang et al., 2015b; Kitai et al., 2016; Senthong et al., 2016). Moreover, elevated TMAO levels in mice exacerbate pressure overload-induced heart failure (Organ et al., 2016) and promote vascular inflammation (Seldin et al., 2016). Consumption of WD has been demonstrated to cause alterations in gut microbiota composition, with decreased levels of Bacteroidetes and Bifidobacteria and increased levels of Firmicutes and Proteobacteria (Turnbaugh et al., 2008; Hildebrandt et al., 2009; Chang et al., 2015). Altered microbiota composition may influence gut microbial TMA production and TMAO synthesis (Chen et al., 2016). Indeed, recent studies have demonstrated that consumption of WD lead to increases in circulating TMAO levels in human (Boutagy et al., 2015a,b). To date, however, no studies have tested whether TMAO plays a role in the pathogenesis of cardiac dysfunction in obesity. In the present study, we investigated the effects of elevated TMAO on the development of cardiac dysfunction in WD-induced obese mice that mimic human obesity syndrome.

## Methods

### Animals

Eight-week-old male CD1 mice were purchased from Vital River (A Charles River Company, Beijing, China). All animals were housed under 12-h light–12-h dark conditions with food and water available *ad libitum*. All experimental procedures and protocols used in this study were approved by the Institutional Animal Care and Use Committee of Jining Medical University, and were performed in accordance with the “Guiding Principles for Research Involving Animals and Human Beings.”

### Protocol

To examine the role of TMAO in cardiac dysfunction in WD-induced obesity, forty mice were fed a normal diet (ND, control) or a WD, simultaneously without or with 1.0% 3,3-Dimethyl-1-butanol (DMB, an inhibitor of TMA formation) in drinking water for 8 weeks (*n* = 10 for each group). This dose of DMB has been demonstrated to effectively inhibit TMA formation and reduce plasma TMAO levels in mice (Wang et al., 2015). The WD (TD 88137, Harlan) contained 42% total fat, 12.8% saturated fat, and 30% sucrose, while ND (Teklad LM-385, Harlan) had 17% total fat, 0.8% saturated fat, and 0% sucrose (Carbone et al., 2015). This WD has been shown to induce cardiac systolic and diastolic dysfunction in mice after 8 week feeding (Carbone et al., 2015). Food intake was measured daily and body weight was measured weekly. Arterial blood pressure and heart rate were measured using a tail-cuff plethysmography (BP-98A; Softron Co, Tokyo, Japan) at baseline and 8 weeks after WD feeding. Cardiac function was assessed by echocardiography at baseline and 8 weeks after WD feeding. At the end of the study protocol, mice were euthanized by cervical dislocation. Blood samples were collected for biochemical analysis. Hearts were rapidly removed and weighed, and the part of the heart was fixed in 10% formalin for analysis of cardiac fibrosis. The remainder of the heart was snap-frozen in liquid nitrogen and stored at −80°C for molecular studies.

### Echocardiography

Cardiac function was assessed using a 15 MHz linear-array transducer, coupled to a Sonos 5500R ultrasonograph (Philips Medical Systems, Andover, MA, USA), as previously described (Carbone et al., 2015; Soliman et al., 2015). Briefly, mice were lightly sedated with 5% isoflurane and placed in the supine position. M-mode and two-dimensional parasternal short- and long-axis scans were obtained to assess changes in left ventricular (LV) dimensions, mass, ejection fraction (EF), and cardiac output (CO). An apical four-chamber view of the left ventricle was acquired, and a pulsed-wave Doppler system was applied to determine the isovolumetric contraction time (ICT) and relaxation time (IRT), the ejection time, and the myocardial performance index (MPI).

### Masson trichrome staining

To assess cardiac fibrosis, Masson Trichrome staining was performed using a Masson Trichrom stain kit (Thermo Scientific, Rockford, IL, USA) following the manufacturer's instructions. Briefly, heart tissues from different groups were sectioned (18 μm) and mounted on glass slides. The sections were placed in Bouin's fluid at 56°C for 1 h and then stained by Biebrich scarlet-acid fuchsin for 10 min. After rinsing briefly in deionized water, sections were placed in phosphotungstic-phosphomolybdic acid solution for 15 min followed by aniline blue staining for 30 min. Sections were then placed in 1% acetic acid solution for 1 min, rinsed, dehydrated, mounted, and imaged under microscope. Cardiac fibrosis was calculated based upon percentages of collagen positive areas in the total myocardial area, as described previously (Wang Y. et al., 2014).

### Biochemical analysis

Plasma TMAO levels were measured using liquid chromatography coupled with triple-quadrupole mass spectrometry as described previously (Ufnal et al., 2014). Plasma glucose levels were measured with a glucose analyzer (Prestige Smart System). Plasma cholesterol and triglycerides levels were measured using commercially available kits (Pointe Scientific, Canton, MI, USA).

### Western blot analysis

Protein levels of pro-inflammatory cytokines tumor necrosis factor (TNF)-α and interleukin (IL)-1β and anti-inflammatory cytokine IL-10 in the heart were analyzed by western blot as previously described (Kesherwani et al., 2015). Briefly, the heart tissue was homogenized in a mammalian tissue lysis buffer with protease inhibitor (Sigma-Aldrich, St. Louis, MO, USA). The protein concentration was quantified by a Bradford assay. Samples were separated on 12% SDS-polyacrylamide gels and then transferred to polyvinylidene difluoride membranes (Millipore Corporation, Bedford, MA). After blocking for 1 h in 5% non-fat dry milk, the membranes were incubated with primary antibodies to TNF-α, IL-1β (Cell Signaling Technology, Beverly, MA, USA), IL-10 (EMD Millipore Corporation, Billerica, MA, USA) and β-actin (Santa Cruz Biotechnology Inc, Santa Cruz, CA, USA) at 4°C overnight. Membranes were then washed and incubated at room temperature with horseradish peroxidase-conjugated second antibody (Santa Cruz Biotechnology Inc, Santa Cruz, CA, USA) for 1 h. Immunoreactive bands were visualized using the enhanced chemiluminescence detection system (Amersham, Arlington Heights, IL, USA) and band densities were analyzed with ImageJ software (National Institutes of Health, Bethesda, Maryland, USA). All data were normalized by β-actin.

### Statistical analysis

Statistical analysis was performed using GraphPad Prism 7 (GraphPad Software, La Jolla, CA, USA). Two-way ANOVA was used to analyze data and differences were analyzed using a multiple-comparison test (Tukey's method). The results are expressed as mean ± SE. Differences were considered significant if *P* < 0.05.

## Results

### Effects of WD and DMB on body weight and metabolic disorders

**Figure 1:**
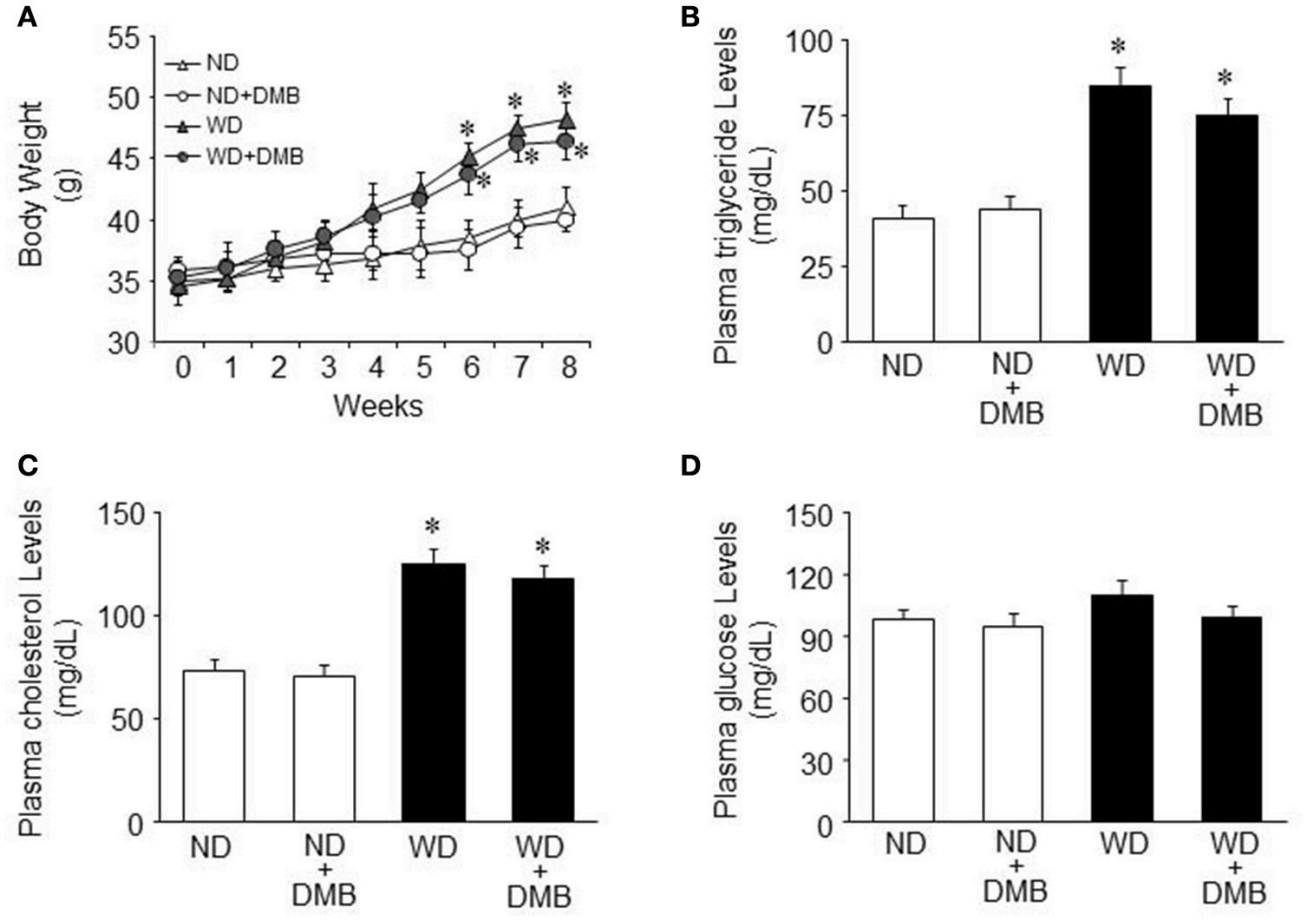
**Effects of western diet (WD) and 3,3-Dimethyl-1-butanol (DMB, an inhibitor of trimethylamine formation) on body weight and metabolic disorders**. Compared with mice fed a normal diet (ND), mice fed a WD for 8 weeks had increased body weight and developed dyslipidemia. DMB treatment had no effects on body weight **(A)** and dyslipidemia **(B,C)**. There were no differences in plasma glucose levels among the four groups **(D)**. Data are presented as mean ± SE (*n* = 10 for each group). ^*^P < 0.05 vs. ND or ND+DMB.

Before WD feeding (baseline), body weight was similar among the four groups (Figure 1A). It was unlikely that there were significant differences in the levels of metabolic parameters before WD feeding, so we did not measure the baseline levels of metabolic parameters.

After WD feeding, mice fed a WD gained significantly more body weight than mice fed a ND after 6 weeks of WD feeding and this trend continued throughout the dietary protocol (Figure 1A). Daily food intake was significantly less (4.3 ± 0.2 vs. 5.5 ± 0.3 g/day, *P* < 0.05) but daily caloric intake was significantly higher (19.8 ± 1.2 vs. 15.1 ± 0.9 kal/day, *P* < 0.05) in mice fed a WD compared with mice fed a ND. At the end of the feeding protocol, the levels of plasma triglyceride and cholesterol were increased in mice fed a WD as compared to mice fed a ND (Figures 1B,C). These data indicate that mice fed a WD for 8 weeks develop obesity and dyslipidemia. An 8 week DMB treatment, beginning at the start of WD feeding, did not alter any of these parameters in mice fed either a WD or a ND. There were no differences in plasma glucose levels across all four groups (Figure 1D).

### Effects of WD and DMB on plasma TMAO levels

**Figure 2:**
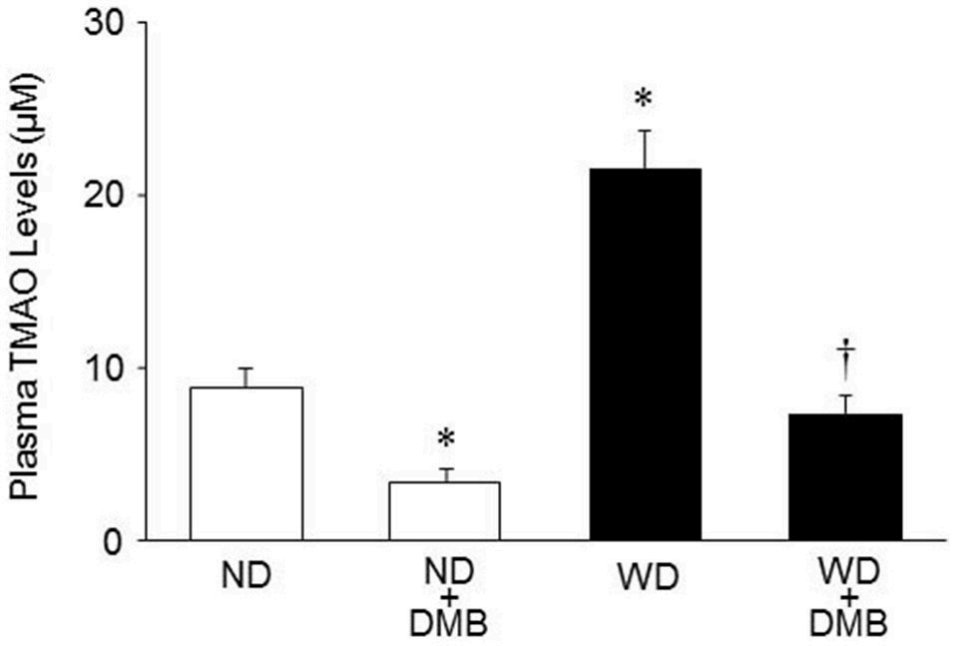
**Effects of western diet (WD) and 3,3-Dimethyl-1-butanol (DMB, an inhibitor of trimethylamine formation) on plasma trimethylamine N-oxide (TMAO) levels**. Mice fed a WD for 8 weeks exhibited increased plasma TMAO levels as compared to mice fed a normal diet (ND). DMB treatment reduced plasma TMAO levels in mice fed either a WD or a ND. Data are presented as mean ± SE (*n* = 10 for each group). ^*^P < 0.05, ND+DMB or WD vs. ND; ^†^*P* < 0.05, WD+DMB vs. WD.

As shown in Figure 2, mice fed a WD for 8 weeks exhibited markedly increased plasma TMAO levels as compared to mice fed a ND. DMB treatment significantly reduced plasma TMAO levels not only in mice fed a WD but also in mice fed a ND.

### Effects of WD and DMB on cardiac function

**Figure 3:**
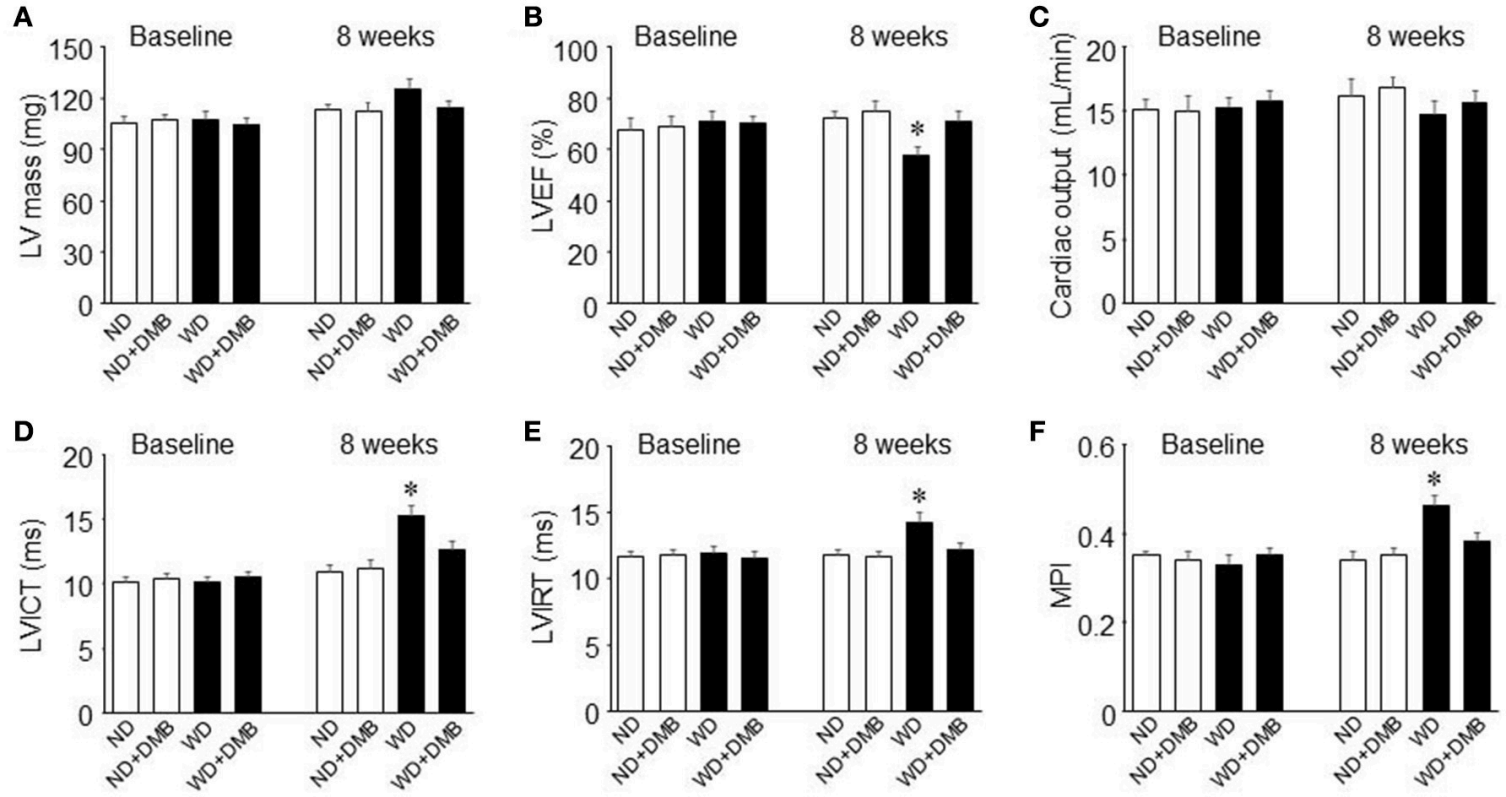
**Effects of western diet (WD) and 3,3-Dimethyl-1-butanol (DMB, an inhibitor of trimethylamine formation) on LV mass (A)**, cardiac systolic **(B,C)** and diastolic **(D–F)** function. Compared with mice fed a normal diet (ND), mice fed a WD for 8 weeks had impaired systolic and diastolic function, which was prevented by DMB treatment. LV, left ventricular; EF, ejection fraction; ICT, isovolumetric contraction time; IRT, isovolumetric relaxation time; MPI, myocardial performance index. Data are presented as mean ± SE (*n* = 10 for each group). ^*^P < 0.05, WD vs. ND or WD+DMB.

Echocardiography showed that there were no differences in LV mass, cardiac systolic and diastolic function across four groups before WD feeding (Figure 3). At the end of the feeding protocol, LV mass was slightly increased in mice fed a WD compared with mice fed a ND, but the difference did not reach statistical significance (Figure 3A). DMB treatment had no effect on LV mass in both groups. Compared with mice fed a ND, mice fed a WD exhibited significantly decrease in LVEF by ~19% (Figure 3B) and increases in LVICT (Figure 3D), LVIRT (Figure 3E) and MPI (Figure 3F) by ~39, 20, and 35%, respectively. DMB treatment prevented WD-induced changes in LVEF, LVICT, LVIRT, and MPI in mice fed a WD but did not alter these parameters in mice fed a ND. No difference in cardiac output (Figure 3C) was observed among the four groups.

### Effects of WD and DMB on cardiac fibrosis

**Figure 4:**
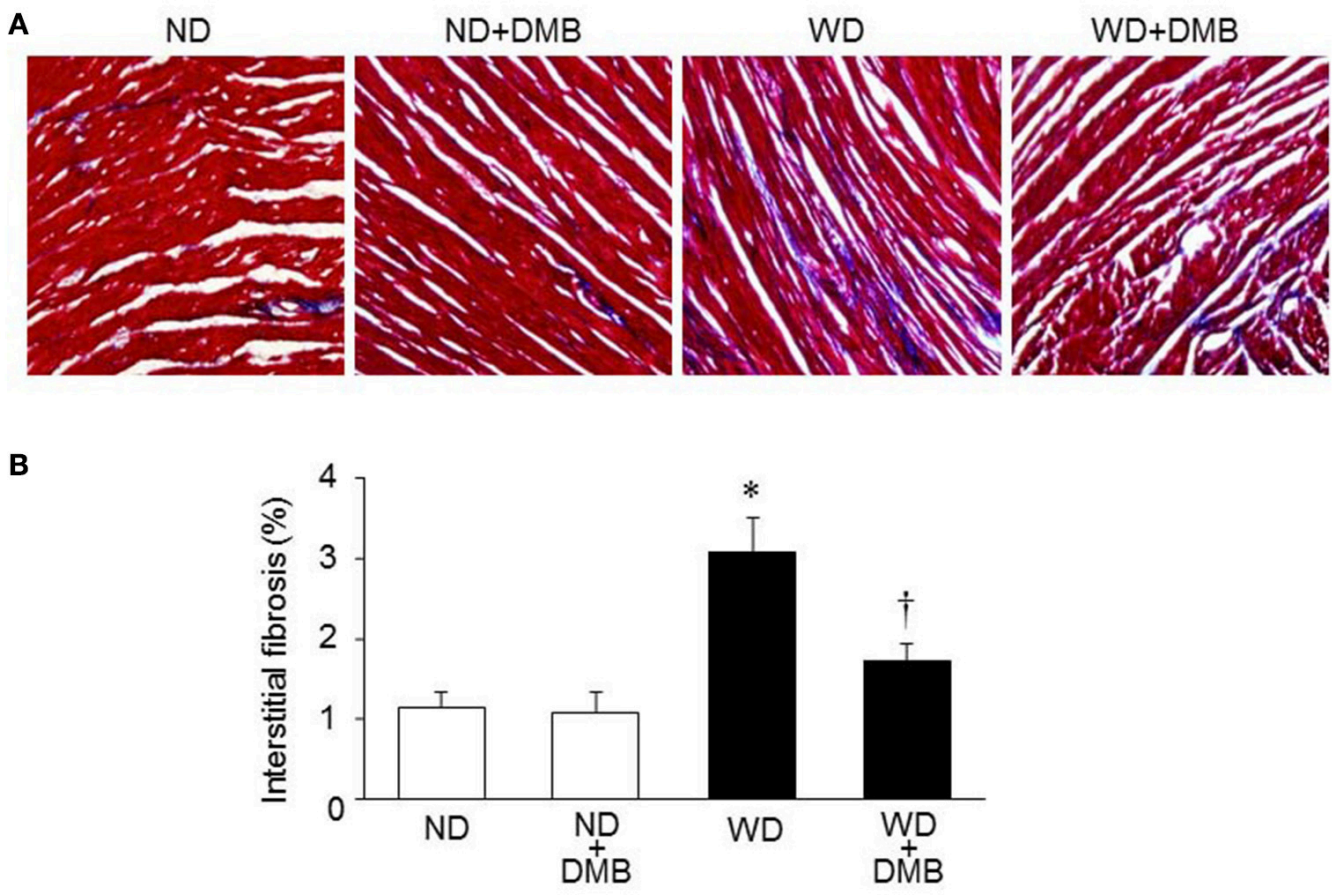
**Effects of western diet (WD) and 3,3-Dimethyl-1-butanol (DMB, an inhibitor of trimethylamine formation) on cardiac fibrosis**. Representative images of Masson Trichrome staining in heart sections are shown in **(A)**. Compared with mice fed a normal diet (ND), mice fed a WD for 8 weeks had increased interstitial fibrosis, which was prevented by DMB treatment **(B)**. Data are presented as mean ± SE (*n* = 10 for each group). ^*^P < 0.05 vs. ND or ND+DMB; ^†^*P* < 0.05, WD+DMB vs. WD.

Increased fibrosis in extracellular tissues has been shown to impair cardiac systolic and diastolic function in animal model of WD-induced obesity (Carbone et al., 2015; Kesherwani et al., 2015). To determine whether elevated TMAO levels contribute to increased cardiac fibrosis in WD-induced obese mice, we performed Masson Trichrome staining to assess cardiac fibrosis at 8 weeks after WD feeding and DMB treatment. We observed significant increase in interstitial fibrosis in mice fed a WD when compared with mice fed a ND (Figure 4). Of note, WD-induced increase in interstitial fibrosis in mice was prevented by treatment with DMB, which had no effect on interstitial fibrosis in mice fed a ND.

### Effects of WD and DMB on expression of inflammatory cytokines in the hearts

**Figure 5:**
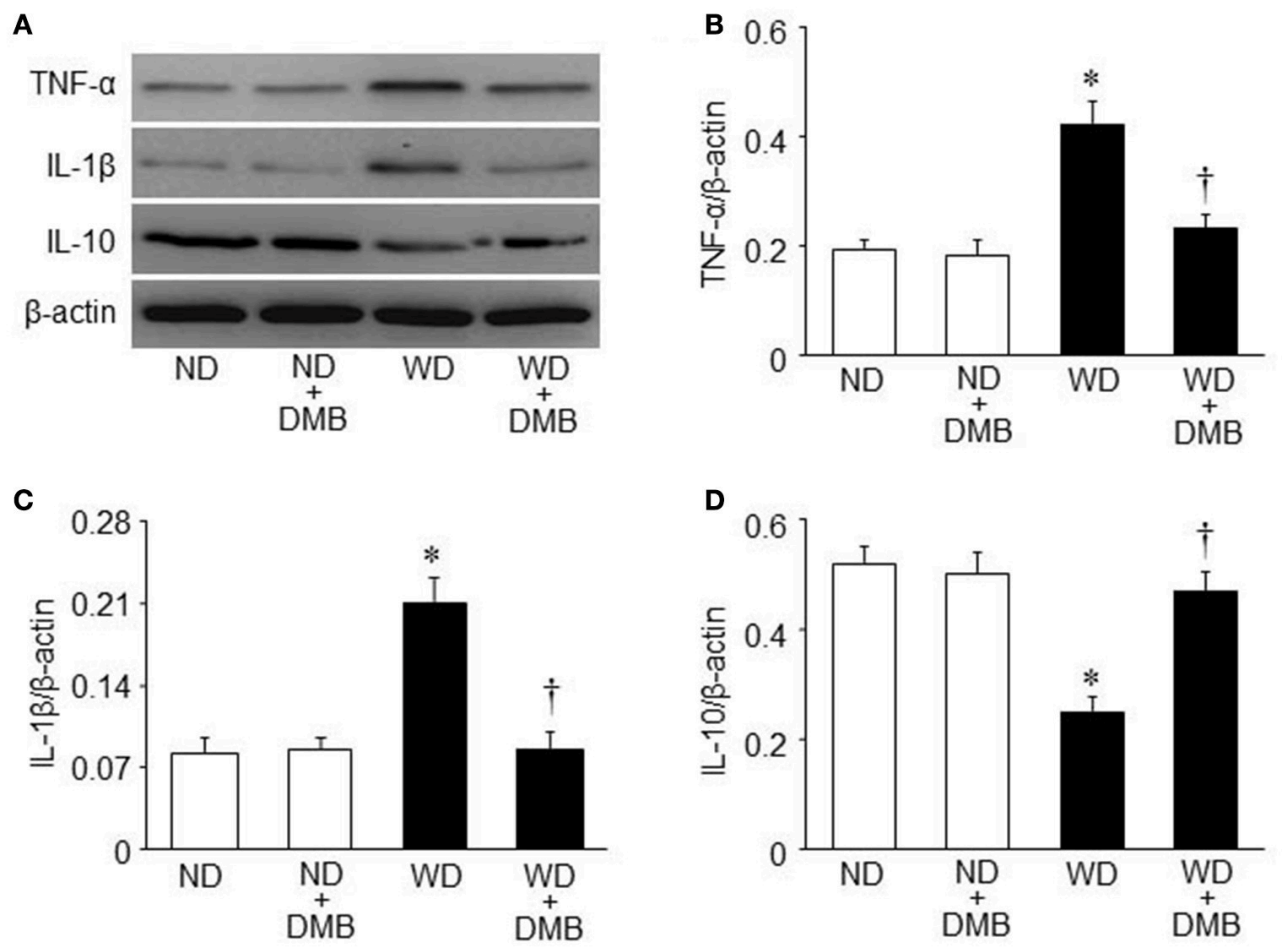
**Effects of western diet (WD) and 3,3-Dimethyl-1-butanol (DMB, an inhibitor of trimethylamine formation) on expression of inflammatory cytokines in the hearts**. Compared with mice fed a normal diet (ND), mice fed a WD for 8 weeks exhibited increased protein levels of pro-inflammatory cytokines tumor necrosis factor (TNF)-α **(B)** and interleukin (IL)-1β **(C)**, and decreased protein levels of anti-inflammatory cytokine IL-10 **(D)**, all of which were prevented by DMB treatment. **(A)** Representative Western blots from each group. Data are presented as mean ± SE (*n* = 10 for each group). ^*^P < 0.05 vs. ND or ND+DMB; ^†^*P* < 0.05, WD+DMB vs. WD.

To further investigate the molecular mechanism by which elevated TMAO levels induce cardiac dysfunction and fibrosis in WD-induced obesity, we measured the expression of inflammatory cytokines in the hearts, which has been demonstrated to be associated with cardiac fibrosis and dysfunction (Sun et al., 2007; Carbone et al., 2015; Kesherwani et al., 2015). As illustrated in Figure 5, an 8 week WD feeding significantly increased protein levels of TNF-α and IL-1β, two key pro-inflammatory cytokines that contribute to cardiac fibrosis, but decreased protein levels of IL-10, an anti-inflammatory cytokine that is involved in the protection against WD or lipopolysaccharides-induced inflammation (Grant et al., 2014). DMB treatment reversed WD-induced changes in protein levels of TNF-α, IL-1β, and IL-10 in the hearts of mice fed a WD, whereas it did not alter protein levels of these inflammatory cytokines in mice fed a ND.

### Effects of WD and DMB on blood pressure and heart rate

**Figure 6:**
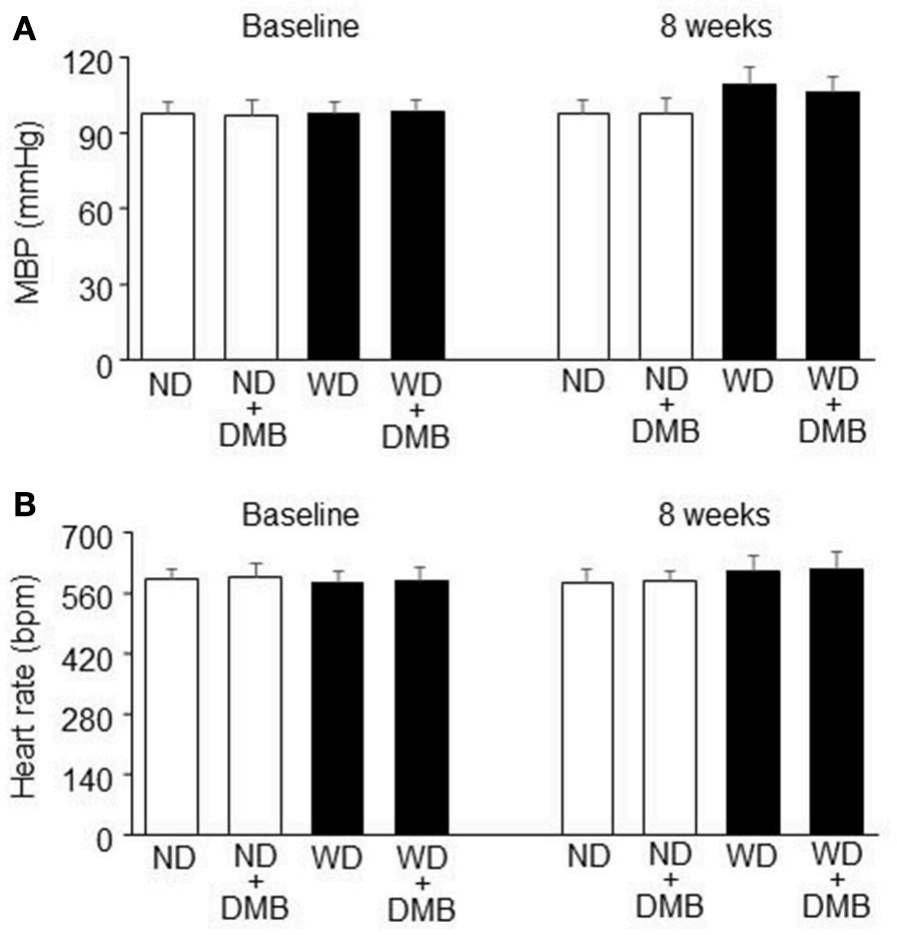
**Effects of western diet (WD) and 3,3-Dimethyl-1-butanol (DMB, an inhibitor of trimethylamine formation) on mean blood pressure (MBP, A)** and heart rate **(B)**. Neither an 8 week WD feeding nor DMB treatment altered MBP and heart rate. Data are presented as mean ± SE (*n* = 10 for each group).

The mean blood pressure tended to be higher in mice fed a WD compared with mice fed a ND after 8 weeks of WD feeding, but there was no statistically significant difference between two groups (Figure 6A). DMB treatment did not affect mean blood pressure in mice fed either a WD or a ND. No difference in heart rate was observed among the four groups (Figure 6B).

## Discussion

The major findings of this study are as follows: (1) compared with mice fed a ND, mice fed a WD for 8 weeks develop obesity and dyslipidemia, and have higher plasma TMAO levels; (2) treatment with DMB, an inhibitor of TMA formation, prevents WD-induced increases in plasma TMAO levels without effects on body weight and dyslipidemia; (3) mice fed a WD for 8 weeks exhibit cardiac dysfunction, which is prevented by treatment with DMB; (4) mice fed a WD for 8 weeks have interstitial fibrosis and inflammation in the heart, which are prevented by treatment with DMB. Taken together, these data suggest that WD feeding increases plasma TMAO levels, which leads to elevated inflammation and interstitial fibrosis in the heart, contributing to cardiac dysfunction.

Cardiovascular disease is the leading cause of death in the world and a known environmental risk factor for the development of cardiovascular disease is WD, a diet rich in sugars and saturated fat (Bäckhed et al., 2004; Foote et al., 2016). Although historically a causal role for WD-induced metabolic disorders has been the primary focus, more recent studies have revealed additional contributory role of dietary-associated and gut microbiota-dependent metabolite TMAO (Brown and Hazen, 2015). TMA containing nutrients including L-carnitine and choline are primarily present in WD. Excessive consumption of WD may influence the capacity of the gut microbiota to generate TMA and, consequently TMAO from these nutrients (Boutagy et al., 2015b). Additionally, consumption of WD can rapidly alter the composition of gut microbiota (David et al., 2014), leading to increased production of TMAO (Kitai et al., 2016). In the present study, we found that mice fed a WD for 8 weeks developed obesity and dyslipidemia but did not significantly increase plasma glucose levels, confirming the results of a previous study (Carbone et al., 2015). Moreover, an 8 week WD feeding significantly increased plasma TMAO levels in mice, which is consistent with the finding of recent studies in human (Boutagy et al., 2015a,b), suggesting that consumption of WD may alter gut microbiota composition and function, and contribute to elevated circulating TMAO levels.

Several studies have shown a strong link between elevated TMAO and cardiovascular disease. For example, examination of sequential subjects (*n* = 1020) undergoing cardiac diagnostic catheterization shows markedly elevated circulating TMAO levels in patients with coronary artery disease (Wang et al., 2011). In a distinct cohort of subjects undergoing cardiac catheterization (*n* = 4007), elevated circulating TMAO levels were shown to independently predict major adverse cardiac events over a 3-year period (Tang et al., 2013). Circulating TMAO levels were also increased in patients with heart failure and these higher circulating TMAO levels were associated with increased 5-year mortality risk (Tang et al., 2014). Moreover, a recent experimental study showed that dietary TMAO directly promotes the development and progression of cardiac dysfunction and heart failure (Organ et al., 2016). Our data showed that mice fed a WD for 8 weeks exhibited cardiac systolic and diastolic dysfunction as evidenced by decrease in LVEF and increases in LVICT, LVIRT and MPI, compared with mice fed a ND. These data confirm previous findings that a WD feeding induces impairment of cardiac function in mice (Carbone et al., 2015; Kesherwani et al., 2015). More importantly, we found that cardiac dysfunction observed in mice fed a WD was prevented by treatment with DMB (an inhibitor of TMA formation), which inhibited WD-induced increases in plasma TMAO levels. Notably, DMB treatment did not alter blood pressure and metabolic disorders in mice fed a WD, thus excluding the possibility that benefic effect of DMB on cardiac dysfunction is due to improvements in blood pressure and metabolic disorders. Collectively, these findings clearly demonstrate that WD-induces increases in circulating TMAO levels independently contribute to cardiac dysfunction in WD-induced obesity.

Increased cardiac fibrosis has been suggested to account for cardiac dysfunction in mice fed a WD (Carbone et al., 2015; Kesherwani et al., 2015). Increased cardiac fibrosis may lead to LV stiffening, loss of compliance and further impairments in cardiac contraction and relaxation (Organ et al., 2016). Consistent with previous findings, we found that cardiac fibrosis was significantly increased in mice fed a WD for 8 weeks, as indicated by Masson Trichrome staining. The increased cardiac fibrosis observed in mice fed a WD was prevented by treatment with DMB, suggesting that increased circulating TMAO levels play a contributory role in mediating WD-induced cardiac fibrosis. Indeed, a previous study has shown that dietary TMAO induces increased fibrosis in an alternative end organ, the kidney (Tang et al., 2015a). In addition, dietary TMAO enhanced renal fibrosis in a manner dose dependently associated with circulating TMAO levels (Tang et al., 2015a). Increased fibrosis was also observed in the hearts of heart failure mice fed a diet supplemented with TMAO and was consistent with enhanced indices of cardiac dysfunction and adverse prognosis (Organ et al., 2016).

Increased cardiac inflammation due to upregulation of pro-inflammatory cytokines and downregulation of anti-inflammatory cytokines has been suggested to contribute to cardiac fibrosis and dysfunction in WD-induced obesity and diabetes mellitus (Sun et al., 2007; Carbone et al., 2015; Kesherwani et al., 2015). To further investigate the molecular mechanism responsible for TMAO-induced cardiac fibrosis and dysfunction in WD-induced obesity, we measured the protein levels of pro-inflammatory cytokines TNF-α and IL-1β and anti-inflammatory cytokine IL-10 in the hearts. We observed significant increases in protein levels of TNF-α and IL-1β and decreases in protein levels of IL-10 in the hearts of mice fed a WD for 8 weeks, which are in agreement with a previous report (Kesherwani et al., 2015). Moreover, we found that DMB treatment prevented WD-induced changes in protein levels for above inflammatory cytokines. These findings indicate that elevated circulating TMAO levels in mice fed a WD induce cardiac inflammation probably by shifting the cytokine profile from anti- to pro-inflammatory cytokines, leading to cardiac fibrosis and dysfunction.

One limitation of this study should be acknowledged. Gut microbiota analysis was not performed in the present study, we did not know how WD influenced the gut microbiota composition in our animal model. Further studies are needed to address this issue by analyzing gut microbiota.

In conclusion, the present study demonstrates that consumption of a WD increases gut microbiota-dependent metabolite TMAO levels in the circulation, which lead to cardiac inflammation and fibrosis, contributing to cardiac dysfunction in mice. The findings from this study provide new insights into the mechanisms underlying cardiac dysfunction in WD-induced obesity. Interventions that reduce circulating TMAO levels may be a novel therapeutic strategy for prevention and treatment of WD-induced cardiac dysfunction.

## Author contributions

Conceived and designed the experiments: KC, XZ, and HZ; Performed the experiments: KC, XZ, MF, and DL; Analyzed the data: KC, XZ, MF, and DL; Wrote the paper: KC, XZ, and HZ.

### Conflict of interest statement

The authors declare that the research was conducted in the absence of any commercial or financial relationships that could be construed as a potential conflict of interest.
